# A metabolic perspective of the neutrophil life cycle: new avenues in immunometabolism

**DOI:** 10.3389/fimmu.2023.1334205

**Published:** 2024-01-08

**Authors:** Mehakpreet K. Thind, Holm H. Uhlig, Michael Glogauer, Nades Palaniyar, Celine Bourdon, Agnes Gwela, Christina L. Lancioni, James A. Berkley, Robert H. J. Bandsma, Amber Farooqui

**Affiliations:** ^1^ Department of Nutritional Sciences, Faculty of Medicine, University of Toronto, Toronto, ON, Canada; ^2^ Translational Medicine Program, The Hospital for Sick Children, Toronto, ON, Canada; ^3^ The Childhood Acute Illness & Nutrition Network (CHAIN), Nairobi, Kenya; ^4^ Translational Gastroenterology Unit, Experimental Medicine, University of Oxford, John Radcliffe Hospital, Oxford, United Kingdom; ^5^ Department of Paediatrics, University of Oxford, Oxford, United Kingdom; ^6^ Biomedical Research Centre, University of Oxford, Oxford, United Kingdom; ^7^ Faculty of Dentistry, University of Toronto, Toronto, ON, Canada; ^8^ Department of Dental Oncology and Maxillofacial Prosthetics, Princess Margaret Cancer Centre, University Health Network, Toronto, ON, Canada; ^9^ Laboratory Medicine and Pathobiology, Faculty of Medicine, University of Toronto, Toronto, ON, Canada; ^10^ Institute of Medical Sciences, Faculty of Medicine, University of Toronto, Toronto, ON, Canada; ^11^ Kenya Medical Research Institute (KEMRI)/Wellcome Trust Research Programme, Centre for Geographic Medicine Research, Kilifi, Kenya; ^12^ Department of Pediatrics, Oregon Health and Science University, Portland, OR, United States; ^13^ Centre for Tropical Medicine and Global Health, University of Oxford, Oxford, United Kingdom; ^14^ Laboratory of Pediatrics, Center for Liver, Digestive, and Metabolic Diseases, University of Groningen, University Medical Center Groningen, Groningen, Netherlands; ^15^ Division of Gastroenterology, Hepatology, and Nutrition, The Hospital for Sick Children, Toronto, ON, Canada; ^16^ Omega Laboratories Inc, Mississauga, ON, Canada

**Keywords:** metabolic reprogramming, neutrophil differentiation, glycolysis, immune mediated diseases, autophagy, mitochondrial respiration

## Abstract

Neutrophils are the most abundant innate immune cells. Multiple mechanisms allow them to engage a wide range of metabolic pathways for biosynthesis and bioenergetics for mediating biological processes such as development in the bone marrow and antimicrobial activity such as ROS production and NET formation, inflammation and tissue repair. We first discuss recent work on neutrophil development and functions and the metabolic processes to regulate granulopoiesis, neutrophil migration and trafficking as well as effector functions. We then discuss metabolic syndromes with impaired neutrophil functions that are influenced by genetic and environmental factors of nutrient availability and usage. Here, we particularly focus on the role of specific macronutrients, such as glucose, fatty acids, and protein, as well as micronutrients such as vitamin B3, in regulating neutrophil biology and how this regulation impacts host health. A special section of this review primarily discusses that the ways nutrient deficiencies could impact neutrophil biology and increase infection susceptibility. We emphasize biochemical approaches to explore neutrophil metabolism in relation to development and functions. Lastly, we discuss opportunities and challenges to neutrophil-centered therapeutic approaches in immune-driven diseases and highlight unanswered questions to guide future discoveries.

## 1 Introduction

**Figure 1:**
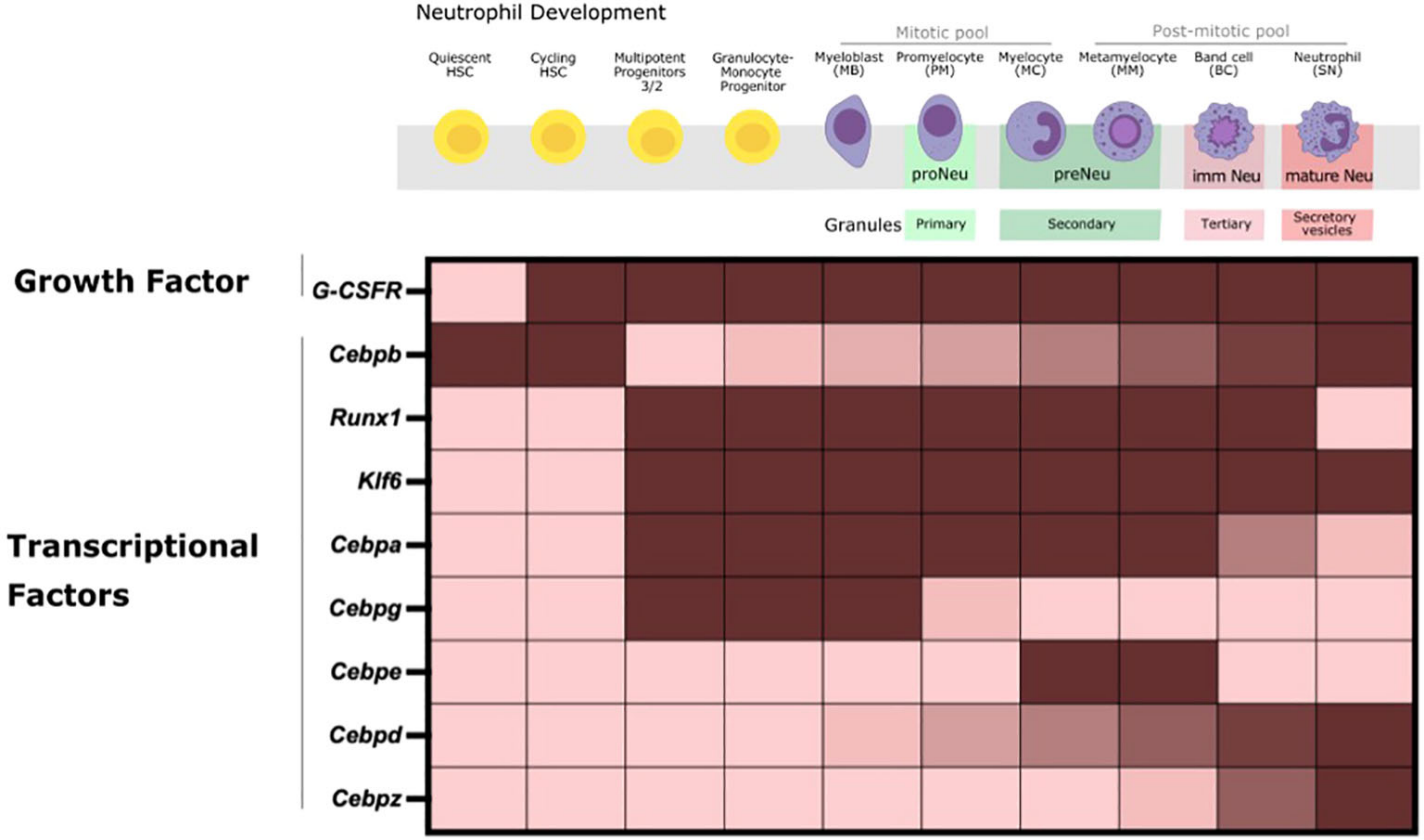
Neutrophil Production in the BM during steady-state happens through multiple successive stages. HSCs proliferate and differentiate into oligopotent committed myeloid progenitors (CMP, GMP) and sequentially into unipotent early neutrophil committed and proliferative progenitors that ultimately give rise to mature neutrophils with the full range of effector functions. Cell- intrinsic regulation of the expression of a multitude of TFs, as depicted here, is crucial to tightly regulate the expression of stage-specific granules (primary, secondary, tertiary and secretory vesicles that contain specific effector proteins) and genes for neutrophil commitment, proliferation, differentiation and maturation. Fully differentiated mature neutrophils then exit the BM to enter into the circulation and tissues under steady-state conditions.

Phagocytic cells have evolved as a key defense line against sterile and microbial insults (1). Neutrophils are the most abundant terminally differentiated effector innate immune cell line in the bone marrow (BM) and peripheral blood. They rapidly localize to sites of infection to implement immediate and effective immune responses for pathogen clearance, and resolution of acute inflammatory responses. In fact, the anti-microbial functions of neutrophils promote an environment that is unfavourable to pathogens at the expense of tissue integrity therefore neutrophil numbers are tightly regulated. Due to their relatively short and variable half-life across tissues (2), neutrophils are constantly replenished from proliferative BM precursors. Differentiation through successive stages maintains their homeostatic levels and ensures their immediate availability to counter invading pathogens (**Figure 1**). Consequently, most hematopoietic stem cells (HSCs) and hematopoietic stem/progenitor cells (HSPCs) in the BM, that give rise to all blood cells through intermediaries, are committed to the production of neutrophils (3, 4). Neutrophils participate in the capture and destruction of invading microorganisms through chemotaxis, phagocytosis, degranulation, reactive oxygen species (ROS) production, formation of neutrophil extracellular traps (NETs), and production of cytokines and other inflammatory mediators upon pathogen recognition [reviewed in (5, 6)]. In this regard, neutrophil activation and subsequent stimulation of specific cell-surface receptors from a broad receptor repertoire (>30) controls neutrophil antimicrobial functions. Defects in neutrophil development, migration, function and clearance increases susceptibility to infection, inflammation and organ dysfunction in multiple organisms (7–10).

## 2 Neutrophil biology: the basics

### 2.1 Neutrophil development from the bone marrow, the circulation and tissues

HSCs and HSPCs in the BM proliferate and differentiate into granulocyte-monocyte progenitors (GMPs). Under the control of granulocyte colony- stimulating factor (G-CSF), produced by macrophages, and endothelial cells (ECs), these GMPs commit to neutrophil generation (granulopoiesis) (11, 12). A pool of hematopoietic progenitors and neutrophil precursors also exists outside the BM, including in the spleen, lung and peripheral circulation, where they differentiate into mature and functionally competent neutrophils to allow acceleration of antimicrobial responses in infected tissues. Notably, neutrophil phenotype reprogramming occurs in tissues regardless of origin (2, 9, 13). During the early phase of an infection and inflammatory response, a rapid increase in *de novo* neutrophil release and production with the appearance of immature neutrophils in the peripheral blood (known as left-shift), termed “emergency” granulopoiesis, helps to meet the enhanced demand for neutrophils as these cells are consumed in large quantities to rapidly counteract bacterial invasion (3, 14–16). This process occurs at the expense of lymphopoiesis and has been reviewed elsewhere in greater detail [reviewed in (14, 17, 18)]. Briefly, conserved microbial elements known as pathogen-associated molecular patterns and host tissue derived damage-associated molecular patterns are recognized by pattern recognition receptors such as, toll-like receptors (TLRs) which are transmembrane proteins, on the surface of multiple immune and non-immune cell types leading to immune cell activation (19, 20). This causes subsequent cytokine and chemokine production and signalling through a common pathway involving nuclear factor kappa-light-chain-enhancer of activated B cells (NF-kB) activation (19). NF-kB, consisting of RelA (p65) and p50, activated downstream of TLRs, which upon phosphorylation by mitogen-activated protein kinases (MAPKs), promote its translocation to the nucleus and leads to robust transcriptional expression of cytokine & chemokine genes including G-CSF, and an array of additional immune genes (19). Dimerization of granulocyte colony-stimulating factor receptor (G-CSFR) by G-CSF, expressed on BM neutrophil precursors (i.e. metamyelocytes and onwards), activates downstream signal transduction pathways [reviewed in (21)]. This regulates HSCs proliferation and differentiation, and directs their commitment and that of early neutrophil progenitors (proNeu1 and proNeu2) (4), and precursors, towards the myeloid lineage (3, 22). This aids in the production of mature neutrophils in order to maintain homeostatic levels under basal and emergency conditions (21, 23). However, G-CSF and G-CSFR independent mechanisms to produce mature granulocytes also exist. This includes IL6-mediated (4) pathways, as well as through intracellular signaling cascades, epigenetic landscapes, and transcriptional networks, such as CCAAT/enhancer-binding proteins (Cebps), to activate specific and tightly regulated gene expression programs for neutrophil differentiation (2). Additionally, there is a role of different nutrient sources, namely glucose, and amino and fatty acids, for HSC lineage specification and commitment (24–26).

As myeloid precursors pass through the various stages of differentiation and maturation to become mature neutrophils in the BM, they undergo morphological changes (nuclear segmentation), increase chemotactic responsiveness and acquire features necessary for microbicidal activity (**Figure 1**). Neutrophil classification based on nuclear morphology, single-cell RNA sequencing (scRNA-seq), and surface marker expression has allowed assessment of mouse and human neutrophil ontogeny, phenotypic heterogeneity and mapping the developmental continuum of cell fate hierarchies (5, 27, 28). The granulopoietic niches of neutrophils within the BM are divided into proliferative mitotic, post-mitotic and the mature neutrophil pool. Neutrophils are released into systemic circulation as a result of differential expression of C-X-C chemokine receptor type 4 and 2 (CXCR4 and CXCR2, respectively) (14, 29, 30). Directional cues from the chemokine stromal cell-derived factor 1, also known as CXC motif chemokine ligand 12 (CXCL12), produced by BM stromal cells, i.e., CXCL12-abundant reticular cells (CAR cells), regulate the CXCL12/CXCR4 chemokine/surface receptor signaling axis for the retention of neutrophils in the BM (31). In contrast, the upregulation of CXC motif chemokine ligand 2 and 1 (CXCL2 and CXCL1, respectively) and CXCR-2 receptor on BM ECs and neutrophils respectively allows for the recruitment of these cells into the circulation, and into naïve and inflamed tissues through activation of downstream signaling pathways (32).

### 2.2 Neutrophil interactions in the tissue microenvironment

#### 2.2.1 Neutrophil crosstalk with innate and adaptive immune cells

It is well accepted that macrophages are responsible for the clearance of apoptotic neutrophils at the end of their lifecycle and resolve inflammation (33). In fact, studies report neutrophils that have physiologically “aged” in the circulation (CD62L^lo^CXCR4^hi^ neutrophils) to be eliminated by macrophages in the BM and tissues. This process also maintains the rhythmic egress of neutrophils into the circulation (30, 34). Similarly, in self-limited lipopolysaccharide (LPS)-induced peritonitis, BM-derived Resolvin D4, a pro-resolving lipid-derived mediator, increases BM-macrophage efferocytosis of apoptotic neutrophils to aid in the resolution infectious inflammation (35). In a mouse model of acetaminophen-induced acute liver injury, neutrophils have crucial functions in liver repair. They do so by promoting the phenotypic conversion of pro-inflammatory Ly6C^hi^CX_3_CR1^lo^ monocytes/macrophages to pro-resolving reparative Ly6C^lo^CX_3_CR1^hi^ macrophages mediated through neutrophil nicotinamide adenine dinucleotide phosphate (NADPH)-oxidase ROS production (36). Neutrophils also support monocyte and macrophage recovery in blood, BM and spleen following genotoxic injury as well T helper 17 cells and macrophage recruitment and priming in a mouse model of atherosclerosis (2, 37). Importantly here, both macrophage or neutrophil-mediated cytokine production leads to immune cell activation and priming for an exaggerated immune response. Therefore the interaction between both is crucial in infectious and inflammatory disease outcomes. These concepts are reviewed elsewhere in greater details (38–40).

A common phenomenon in chronological ageing known as reverse transendothelial migration (rTEM), where neutrophil exhibit retrograde mobility within ECs junctions and re-enter the vascular lumen. This process is shown to be mast cell-derived CXCL1 dependent (32, 41, 42). Here, the rTEM neutrophils are of noxious phenotype capable of inducing remote organ damage in acutely inflamed aged tissues. In fact, multiple studies also show neutrophil recruitment in physiology to be mast cell dependent (43–46). The impact of neutrophils on T cells in various disease contexts (47–52) is also summarized in excellent reviews (39, 40, 53–55).

#### 2.2.2 Neutrophil crosstalk with non-immune cells

Neutrophils’ interaction with non-immune cells is crucial to regulate their biology. In this regard, BM CAR cells, a population of mesenchymal stem cells, produce CXCL12 that mediates the retention of neutrophils in the BM through CXCR4 ligation (31). Platelet-neutrophil interaction are also implicated in homeostasis and inflammation (40, 54, 56–59). Neutrophil and ECs interaction allows for unidirectional migration through venular walls (54, 60, 61). Most importantly, aberrant neutrophil-endothelial interactions are implicated in a wide range of inflammatory diseases that relate to neutrophil influx and tissue damage (62–64). Similarly, neutrophil-epithelial crosstalk is involved in the maintenance of the epithelial-lined organs where uncontrolled neutrophil processes contribute to pathogenesis of diseases (65–68).

### 2.3 Mechanisms implicated in neutrophil-mediated immunity

Acute and chronic inflammatory and infectious immune responses are marked with heightened immune cell recruitment, and dramatic shifts in tissue and systemic metabolism including nutrient depletion, hypoxia and the generation of large quantities of reactive nitrogen and oxygen intermediates (69). There is an heightened interest in identifying the role of unique metabolites and metabolic pathways in immunoregulation, a field termed immunometabolism, ranging from energy metabolism to the modulation of signalling pathways and post-translational modifications (70–74). Traditionally, neutrophils are thought to be purely glycolytic and the role of mitochondria, a central organelle for energy homeostasis and metabolic control, is believed to be minimal, for neutrophil function (75). However, this view has been challenged, as their critical role as first line defenders requires high metabolic plasticity to respond to environmental cues and regulate innate immune responses (76).

#### 2.3.1 Overview of neutrophil metabolism

Circulating neutrophils primarily rely on glycolysis and the pentose phosphate pathway (PPP), both of which take place within the cytosol, as their preferred metabolic strategy to fuel phagocytosis, ROS production, and NET formation. However, fatty acid oxidation (FAO), tricarboxylic acid (TCA) cycle, and oxidative phosphorylation (OXPHOS) that occur within the mitochondria, are also undoubtedly crucial under both steady-state and inflammatory conditions (77–80). Although immunometabolism (70, 81) and metabolic plasticity is increasingly understood, minimal data is available on neutrophil metabolic reprogramming under diverse nutritional, metabolic and pathologic conditions. This review is therefore focused on key neutrophil functions and their dependence on different key metabolic pathways in experimental animals and human cohorts. We also emphasize approaches used to study immunometabolism and how different nutrient environments contribute to neutrophil biology.

## 3 Metabolic programmes for granulopoiesis

Neutrophil production is tightly regulated by intrinsic and extrinsic cellular factors via a number of transcription factors (TFs) that regulate subsequent stages of neutrophil development. The hierarchical expression and activation of tightly defined TFs is required for lineage specification of HSPCs and proper commitment and differentiation of myeloid precursors into mature neutrophils in the BM (**Figure 1**) (9, 31). TFs are largely regulated at transcriptional, translational, and posttranslational levels. Consequently, transcriptional control, isoform usage, phosphorylation, and acetylation of TFs are crucial for the proper activation of gene regulatory mechanisms that orchestrate the differentiation of HSCs into committed cells.

### 3.1 Transcriptional regulation of granulopoiesis

TF such as Cebps and others work in a combinatorial manner to orchestrate the transcriptional networks for neutrophil lineage commitment, proliferation, differentiation and functional responses. The expression of these TFs is also correlated with stage-specific granule expression across the neutrophil lineage and therefore antimicrobial capacity (4, 23, 82, 83). Changes in its expression can significantly affect downstream genes that impact the neutrophil development and function trajectory (84).

Cepba binds to promoters of myeloid-related genes to activate myeloid-lineage gene expression program and repress non-myeloid lineage genes in hematopoietic progenitors of multi-lineage potential in the BM. The combinatorial expression of Cebpa, Gata1 and low levels of PU.1 are required at the GMP stage for granulopoiesis initiation, and expression of primary granule proteins, and Cebpa deletion is shown to skew progenitors towards lymphopoieses (31, 81, 85, 86). Similarly, Cebpg is a pro-proliferative factor that is particularly required by myeloblasts, promyelocytes and myelocytes. Cebpe further drives differentiation at myelocytes, and metamyelocytes and contributes to the expression of secondary granules (4, 31). Here, Cebpe deletion leads to an accumulation of GMPs (4, 31). Expression of Cebpb, Cebpd, and PU.1 is found in the most mature neutrophil precursors and in peripheral blood granulocytes. However, studies in Cebpb deficient mice do not observe defects in steady-state granulopoiesis, but rather Cebpb is shown to be crucial for “emergency” granulopoiesis (86, 87). On the contrary, Cebpd and PU.1 are involved in the expression of tertiary granules such as matrix metalloproteinase-9 (Mmp9) and CXCL2 at the band cell stage for the terminal differentiation of neutrophils (31, 81). The expression of Cebpz is prominent from band cell onwards with peak levels in the most mature neutrophil precursors and in peripheral blood granulocytes (31, 81). Additionally, Runx1 and Klf6 are shown to modulate neutrophil maturation (9).

Altogether, co- expression of specific TFs within specific subpopulations serves to drive early lineage specification towards distinct progenitors. These TFs also function during the later developmental stages to drive differentiation and maturation for optimal antimicrobial defense. Newer studies focus solely on the global gene expression patterns of these central TFs to drive neutrophil ontogeny through bulk and scRNA-seq to assess transcriptional programs across neutrophil subsets (31, 81, 88). However, the post-transcriptional modifications and mechanisms that enable these transcriptional signals to regulate coordinated neutrophil differentiation remain incompletely understood. Nevertheless, there is some evidence on the role of changes in genome accessibility, master transcriptional regulators and metabolites for controlling these TFs and subsequently immune responses, some of which are discussed in more detail below (9, 23, 23, 73).

### 3.2 Metabolic regulation of the transcriptional regulatory networks

An intricate link between cellular metabolism, transcription, and signalling pathways directly and indirectly supports the regulation of genes involved in cellular differentiation and cellular processes (73, 81, 84, 89). It is well-accepted that the mitochondria, through TCA metabolites, control chromatin modifications, DNA methylation, and post-translational modifications of proteins to alter their function. However, how specific metabolites regulate TFs for neutrophil development has not been described previously (73, 90). Enhancing intracellular nicotinamide adenine dinucleotide (NAD^+^) levels, through Vitamin B3 supplementation, is found to support sirtuin-1, Cebpa, Cebpb, G-CSF and G-CFSr expression for neutrophilic differentiation and migration in CD34+ hematopoietic progenitor cells, the promyelocytic leukemia cell line HL-60, and in primary bone marrow CD34+ cells from severe congenital neutropenic patients (91). Glucose metabolism has also been shown to regulate Cebpb activity for adipogenic gene expression and differentiation of preadipocytes to adipocytes (92). Glucose can induce nicotinamide mononucleotide adenylyltransferase (NMNAT)-2 expression, an enzyme for NAD^+^ biosynthesis, and reduce nuclear poly (ADP-ribose) polymerase (PARP)-1 enzymatic activity with Cebpb. Conversely, glucose deprivation reduces NMNAT-2 levels, increases the enzymatic activity of PARP-1 and reduces the binding of Cebpb to target gene promoters thereby preventing adipogenesis (92). At the same time, activity of mammalian target of rapamycin complex (mTORC)-1 controls Cebpb expression and its activity is inhibited under caloric restriction (93). The mechanism of Cebpb regulation by glucose, NAD+, and mTORC1 remains unknown. The Vitamin D receptor and Retinoic Acid Receptor α in human acute myeloid leukemia cell lines (HL60, and KG1 cells) is also shown to regulate the expression of Cebpa, Cebpb and subsequently Cebpe for granulocytic differentiation (88). While these studies examine the direct and indirect roles of metabolites for Cebp regulation in the adipose, liver tissues and specific cell lines, the metabolic pathways regulating these TFs in the hypoxic BM microenvironment for neutrophil development remains an open avenue for future research. A study of autophagy Atg7 deficient neutrophils demonstrated that the metabolic program does not influence the transcriptional program in neutrophil precursors where these precursors are morphologically and functionally immature (7). Therefore, further studies are needed to identify the parallel roles of transcriptional regulators and metabolic programming, if any, for cellular differentiation. Interestingly, glycolytic and mitochondrial metabolites are shown to control chromatin modifications, and DNA methylation in physiology and disease and may be relevant in this context (73, 94).

### 3.3 Metabolic programming of cellular differentiation

#### 3.3.1 Metabolism as a key determinant of hematopoietic stem cell fate

**Figure 2:**
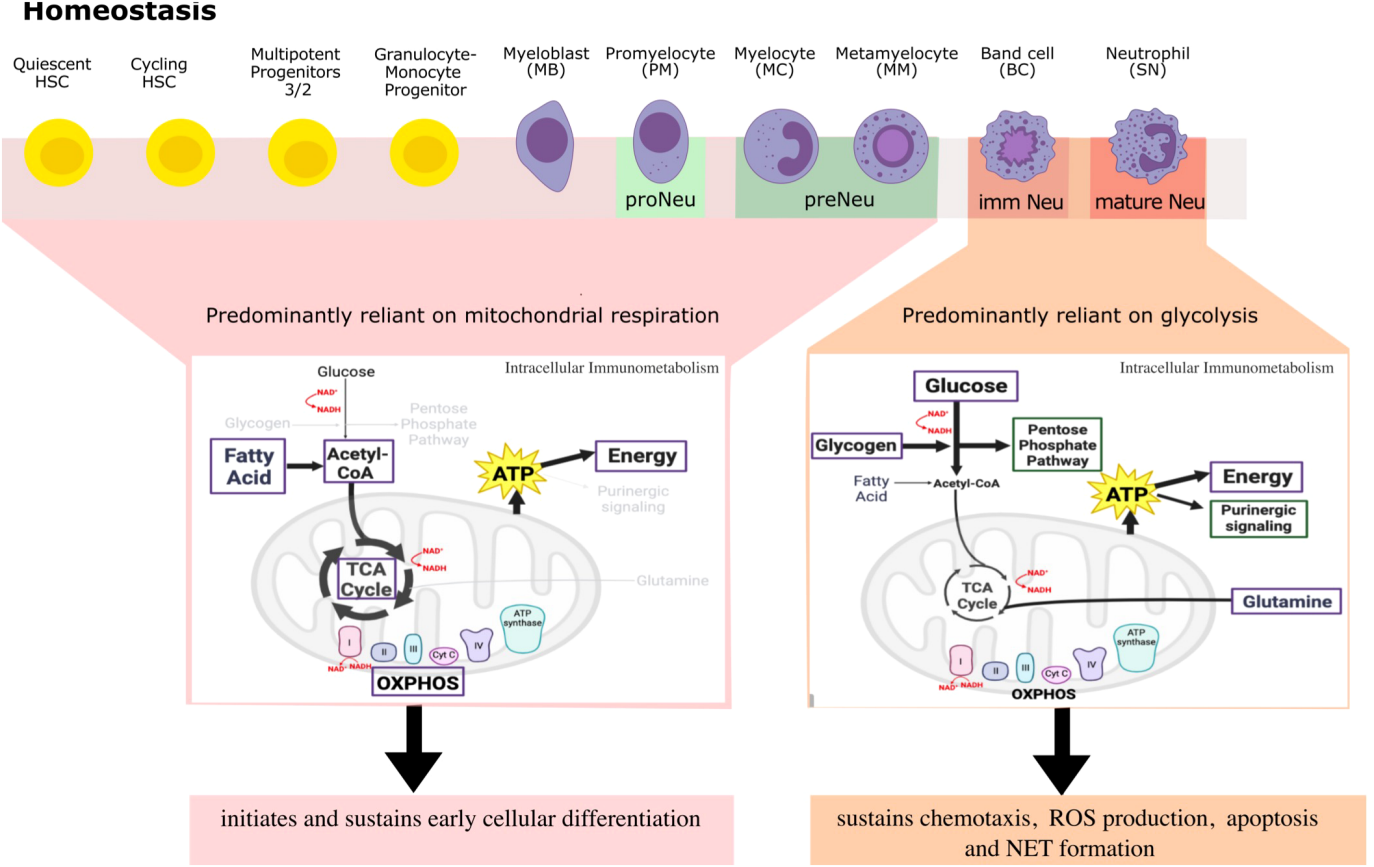
A simplified overview of differential metabolites and metabolic pathways that neutrophils engage in throughout differentiation and beyond to regulate their large repertoire of immune responses in steady-state. Here, neutrophil committed progenitors and early precursors engage in fatty acid oxidation, TCA cycle and OXPHOS for ATP generation that sustains neutrophil differentiation programs. Indeed, higher mitochondrial activity is indicative of a more immature neutrophil phenotype. Mature neutrophils on the other hand, rely primarily on glycolysis and little mitochondrial respiration to regulate and sustain ATP production for effector functions and for autocrine purinergic signaling. Metabolic requirements of neutrophils are not ‘black and white’ in inflammatory conditions, but rather a continuum of these processes works together to regulate an optimal response, a perspective that requires more investigation. Highlighted in purple are metabolites used for energy production and green are pathways used by metabolites to regulate neutrophil functions.

Differentiation of HSCs into fully functional mature neutrophils requires considerable energy for extensive cytoplasmic and nuclear remodelling (95) (**Figure 2**). Autophagy decreases mitochondrial mass in quiescent HSCs for their self-renewal capacity. As a result, quiescent HSCs primarily rely on anaerobic glycolysis to maintain stem cell quiescence and self-renewal in the low oxygen niches of the BM. Increased peroxisome proliferator-activated receptor δ (PPAR δ ) activation for mitochondrial biogenesis, mitochondrial ROS, and FAO in cycling HSCs demarcates asymmetric division, one daughter with stem cell features and one committed progenitor, and further regulates HSC maintenance (25, 71, 78, 96–100). Therefore, the metabolic programs of HSCs balance self-renewal and commitment. Similarly, a lower mitochondrial mass and mitochondrial activity and increased transcriptional activation of the TF hypoxia-inducible factor 1-alpha (HIF-1a) to drive aerobic glycolysis by Meis1 in quiescent HSCs, allows for a long-term reconstitution capacity of these cells in transplantation experiments. Here, increased mitochondrial biogenesis is associated with exit from quiescence (25, 26, 97, 98, 101). In fact, flow cytometric analysis of the metabolic profile of HSCs using tetramethylrhodamine methyl ester (TMRM) shows only 6%-9% of total BM cells with low mitochondrial potential (TMRM^lo^) but this population contains more than 80% of quiescent HSCs (98). Interestingly, TMRM^lo^ quiescent HSCs cultured for 5 days under differentiation-inducing conditions (with cytokines SCF, Flt3, IL-3 and IL-6) and carbonyl cyanide-p-trifluoromethoxyphenylhydrazone (FCCP) revert to a state of self-renewal. TMRM^hi^ quiescent HSCs are however unable to revert to functional stem cells (25). Additionally, proteomic analyses highlights the role of increased translation of mitochondrial transcription factor A (TFAM) in proerythrocytes and its regulation of downstream genes associated with mitochondrial metabolism for proper erythrocyte differentiation (102). Similarly, to improve the efficacy of hematopoietic cell transplantation, where limited numbers of HSCs are present, transplantation of CD34+ HSCs with peroxisome proliferator-activated receptor γ (PPARG)-specific small hairpin RNA (shRNA) can promote expansion of HSCs and HSPCs. This is through enhanced glycolysis and HSC self-renewal, conversely inhibition of glycolysis and enhancement in PPARG suppresses this expansion (95, 103). These studies highlight the essential role of nutrients and their associated signaling pathways on the self-renewal, differentiation and lineage commitment potential of HSCs.

#### 3.3.2 Metabolism as a master regulator of neutrophil state

The metabolic state also shapes committed progenitor cells. Expression of type III receptor tyrosine kinase ckit, in early committed and immature neutrophil progenitors in the BM requires enhanced mitochondrial function, i.e., oxidative phosphorylation, to promote rapid symmetric differentiation. This further highlights the essential role of increased mitochondrial activity for HSC cell fate decisions under steady-state (71, 104). Similarly, transcriptomics and proteomics has shown that mitochondrial function is essential in the early stages of granulopoiesis for lineage commitment and the initiation of neutrophil differentiation in ckit^+^ neutrophils. Mitochondrial function is key for the differentiation in ckit^+^ HSCs from free fatty acid utilisation (104–106). Complex III of the electron transport chain may be responsible for ATP generation in early myeloid precursors in the BM in this context (47, 105). Similarly, shRNA-mediated knockdown of alanine, serine, cysteine transporter 2 (ASCT2) glutamine transporter in CD34^+^progenitors, upregulates CD11b myeloid marker, highlighting the skewness of the progenitors towards the myeloid lineage that happens irrespective of glucose and glutamine metabolism (71). Adenylate kinase 2 (AK2) regulates the homeostasis of mitochondrial adenine nucleotides (ADP, ATP and AMP) by catalyzing the transfer of high-energy phosphate in the mitochondrial intermembrane space, once the HSCs commit to a specific lineage pathway. AK2 deficiency results in impaired proliferation and differentiation in granulocyte precursor cells shown using the HL-60 promyelocytes cell line, through impairment in oxidative phosphorylation for energy metabolism (107). In fact, even though enhancement of glycolysis is evident by accumulation of pyruvate and lactate, neutrophil differentiation is hindered due to incomplete mitochondrial activity by hematopoietic progenitors that is required to meet the greater energy demand. This suggests an indispensable role of mitochondrial metabolism in neutrophil differentiation (106, 107). This is relevant for reticular dysgenesis, an inherited immune deficiency disorder caused by AK2 deficiency. As neutrophils differentiate and become mature neutrophils, there is a decline in mitochondrial number and activity with a change in mitochondrial morphology from a more tubular phenotype to round to regulate spontaneous cell death (108–110).

#### 3.3.3 Role of autophagy for mitochondrial metabolism in neutrophil differentiation

Autophagy and mitochondrial respiration are critical determinants of neutrophil differentiation. Autophagy is a conserved cellular recycling process, involving several conserved autophagy-related genes (*Atg*), that enables the degradation of cytoplasmic content in lysosomes for diverse cellular processes. In fact, autophagy (regulated by *Atg5* or *Atg7*) degrades lipid droplets, providing free fatty acids used by the mitochondria through the TCA cycle and oxidative phosphorylation for ATP production at the myeloblast and myelocyte stage for terminal neutrophil differentiation (**Figure 2**) (7). As a result, higher oxygen consumption rate (OCR) is evident in BM neutrophils. Here, microfluidic gene expression analysis with the Fluidigm-Biomark array reveals that all 15 glycolytic pathway genes analyzed are downregulated while the mitochondrial content simultaneously undergoes a 2-fold increase during normal neutrophil differentiation (47, 111). This is further validated by targeted metabolomics. In *Atg7* deficient myeloblast cells, ECAR (measure of glycolysis, extracellular flux assay) and lipid accumulation, assessed through lipidomics, is increased, and mitochondrial ATP production is decreased. As a result, the expression of *Ldha* is also increased to generate lactate and NAD*^+^* to keep glycolysis going but the ATP levels and differentiation is not restored to wild-type amounts (7). In the same study, administration of either free fatty acids or pyruvate is able to restore energy metabolism and differentiation, supporting that mitochondrial respiration is essential for early neutrophil differentiation where energy requirements are the highest (7). Furthermore, deletion of *Atg7* at the GMP stage also leads to failure of neutrophil differentiation and an accumulation of immature forms which are functionally defective. Deletion of autophagy genes at later development stages results in a different phenotype where mature neutrophil are primarily expressed (7, 112). This reflects the need to carefully consider the developmental stages where autophagy genes are knocked down as it may have differential effect on neutrophil maturation.

Conflicting data indicates that since neutrophil mitochondria have decreased protein expression of some complexes, they may not be fully involved in ATP production but rather in maintaining functional responses through the mitochondrial membrane potential ( ΔΨmt ) (105, 113). In fact, studies show that inhibition of complex III in both glucose-rich and glucose-depleted environments, completely diminishes ΔΨmt  but without an effect on ATP production. This suggests a role for mitochondria that extends beyond oxidative phosphorylation and ATP production for neutrophil development (105, 109).

## 4 Metabolism driven regulation of neutrophil migration

### 4.1 Neutrophil mobilization and trafficking

Neutrophil mobility is essential for cell recruitment to sites of inflammation and infection. Chemotaxis, is the ability of neutrophils to sense gradients, polarize and directionally migrate within a chemotactic gradient field to access invading pathogens [reviewed in (114, 115)]. TFs KLF6 and RUNX1 are shown to be valuable for controlling genes for neutrophil migration into inflamed sites (9). Interestingly, “swarming”, tightly regulated by exosomes and lipid leukotriene 4 (LTB4), is also the coordinated feedforward movement of many neutrophils to accumulate in large numbers in inflamed and infected tissues in early stages of infection for optimal bacterial clearance (116–118). This is followed by migration arrest at later stages of infection to avoid uncontrolled aggregation at inflamed sites through G protein-coupled receptor kinase 2 (GRK2)-mediated G protein-coupled receptors (GPCR) desensitization, and prostaglandin E2 (PGE2) synthesis to promote anti-inflammatory programs for tissue repair (119, 120). Resolvin D4 also mediates this migration arrest (35).

In contrast, transendothelial migration (TEM) defines neutrophil migration across the endothelium to both inflamed and naïve sites, albeit at lower levels. This is dependent on receptor ligand interactions of adhesins, integrins and selectins. Adhesion molecules i.e., chemokines, integrin ligands and selectins on the endothelium, bind to G-protein-coupled chemokine receptors, integrins (Mac1-CD11b/CD18), and selectin ligands on the surface of neutrophils. These molecules tightly regulate rolling, adhesion and transmigration, i.e., paracellular (~96% of the time) vs. transcellular into tissues, as has been well defined in excellent reviews [reviewed in (113)]. Additionally, rTEM, re-entry of activated neutrophils from inflamed sites into the circulation, due to overexpression of CXCL1 at endothelial junctions and CXCR2 desensitization in mice also controls inflammatory responses (41, 42).

### 4.2 Metabolic regulation of neutrophil trafficking

**Figure 3:**
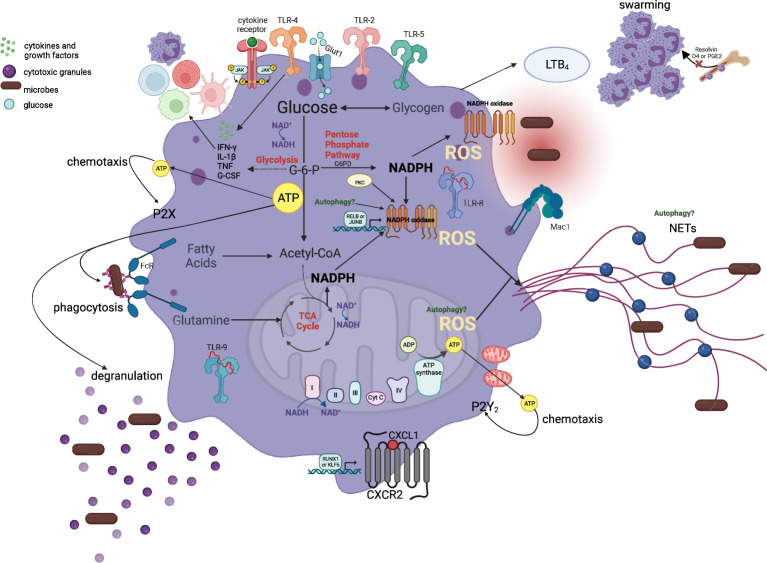
Metabolic pathways involved in the maintenance of neutrophil effector functions. Neutrophils rely primarily on glycolysis and very little mitochondrial respiration to regulate and sustain ATP production for chemotaxis, phagocytosis, cytokine expression, ROS production, degranulation, and NET formation in the circulation and peripheral tissues. An initial burst of mitochondrial-derived ATP release and a second phase that involves glycolytic ATP production maintains the autocrine purinergic signaling for chemotaxis. Glucose uptake and glycolysis regulates phagocytosis and degranulation while PPP is indispensable for NADPH generation, which is oxidized by the NADPH oxidase for ROS generation and eventually NETs. Additionally, mitochondrial-derived ROS can also enhance NET production. NADH/NAD ratio is crucial for the maintenance of glycolytic and mitochondrial ATP production and thus functional properties of neutrophils. Multiple metabolites, as depicted in this figure, including glycogen, and glutamine, are also capable of entering these metabolic pathways to regulate and sustain neutrophil responses in tissues. This metabolic flexibility of neutrophils allows them to coordinate optimal effector responses in tissues with diverse substrate availability.

Metabolic stress is a hallmark of several conditions including trauma and infection. Neutrophils isolated from trauma patients have enhanced markers of mitochondrial damage, i.e., mitochondrial-derived damage-associated molecular patterns, inflammasome activation, and reduced expression of CXCR2, thereby impaired chemotaxis (121, 122). Studies further show that upon stimulation of neutrophil-like HL-60 cell line with N-formyl-methionyl-leucyl-phenylalanine (fMLP), which activates formyl peptide receptors (FPRs) expressed on these cells, mitochondria with high ΔΨmt localize to cell protrusions and mitochondrial-derived ATP is released into the extracellular spaces. This serves as an autocrine messenger to amplify chemotaxis signals through activation of P2Y_2_R-mediated mTOR signaling at the leading edge, rather than as a direct energy source (123). In fact, two phases of purinergic signaling are suggested: an initial burst of ATP release that is driven by mitochondrial ATP and a second phase that involves glycolytic ATP production to maintain chemotaxis (**Figure 3**) (123). In this study, the fluorescent ATP probe 2-2Zn(II) anchored on cell membrane of live neutrophils reveals an increase in extracellular ATP production by the mitochondria immediately upon stimulation of cells with fMLP (123). Similarly, pretreatment with carbonyl cyanide 3-chlorophenylhydrazone (CCCP) or FCCP for 2 hours shows a direct relation between loss of ΔΨmt , detected by increased green fluorescence of JC-1 probe, rounded cell shape and the inhibition of chemotaxis (75). Studies in zebrafish also show that neutrophils depend on ΔΨmt for chemotaxis and that neutrophil-specific disruption of mitochondria *in vivo* is associated with inhibited motility, with an inability to reduce ROS species, and cell apoptosis (**Figure 3**) (124, 125). Similarly, hyperglycemic conditions in cancer provide microenvironments that promote metastasis of tumor-associated neutrophils and impair mobilization of antitumor neutrophils, resulting in poor prognosis that is seen for these patients. This shows that migration is selective based on the dominant neutrophil subset in the disease state and their preferred fuel of choice (126).

## 5 Modulation of metabolism regulates NADPH-oxidase dependent functions

### 5.1 Overview of NADPH-oxidase dependent antimicrobial functions

As myeloid precursors become mature neutrophils, they acquire features necessary for optimal microbicidal activity including stage-specific granule and NADPH oxidase proteins (**Figure 1**), the most abundant proteins in mouse and human neutrophils and the main complex for neutrophil ROS production, respectively (31, 81, 127, 128). In fact, primary [cathepsin G (CTSG), myeloperoxidase (MPO), elastase (ELANE)], secondary [lactoferrin (LTF), neutrophil gelatinase-associated lipocalin (NGAL), cathelicidin (CAMP)], and tertiary (MMP9, lipocalin 2 (LCN2), CD11b/CD18, CXCL2) granules and secretory vesicles (β2 integrins), are well-defined granules that carry distinct types of cargo as mentioned. These highly toxic granule proteins are released into the phagocytic vacuole or extracellularly in a ‘formed-first-released-last’ model to help destroy invading pathogens (69, 128). In this context, the strength of the stimulus initiates the release of granules, both intracellularly and extracellularly, with the secretory vesicles being mobilized first and primary granules secreted at the end in response to multiple inflammatory mediators (129).

The NADPH oxidase (NOX) is a multicomponent electron-transfer complex that generates superoxide and other ROS into the phagosome or extracellularly for microbial killing events (23). This complex is comprised of five subunits that are expressed at different stages during neutrophil differentiation and packaged into the granules. More specifically, p22, p40, and Rac1/2 have stable gene expression levels throughout all stages, whereas gp91 has maximal gene expression at the metamyelocyte and band cell stages, and p47 and p67 are upregulated in the metamyelocytes and have the maximal gene expression at the mature stages (23). Consequently, studies show that while all NOX subunits are expressed by the metamyelocyte stage, peak levels of ROS are only reached at the end of differentiation (23). Interestingly, RELB and JUNB are important TFs for the expression of these genes for NADPH ROS generation (9). In steady-state, the membrane-bound (b558 - gp91, p22) and cytosolic components (p47, p67, p40, Rac1/2) do not interact with each other. Upon phagocyte activation in mature neutrophils, consumption of oxygen is increased, and these subunits are phosphorylated by protein kinase C (PKC). The cytosolic components migrate almost instantly to the phagocyte membrane where they assemble with the flavocytochrome b558 to form the active enzyme. In this regard, NADPH-oxidase mediated ROS production accounts for majority of neutrophil ROS production (127). Excessive ROS production can also be damaging to the host cells therefore, balanced levels of ROS are crucial for pathogen clearance and restoration of tissue homeostasis.

### 5.2 Metabolic reprogramming for pathogen killing via NADPH oxidase ROS production

In this section, we aim to summarize the literature as it relates to metabolic requirements for neutrophil ROS generation.

#### 5.2.1 Pentose phosphate pathway

When glucose enters cells, it is phosphorylated by hexokinase generating glucose-6-phosphate (G6P). G6P can flow through two different pathways: the glycolytic pathway, generating fructose-6-phosphate that is metabolized to lactate, to produce ATP, or the pentose phosphate pathway (PPP) that converts NADP+ to NADPH (**Figure 3**) (130–132). The latter is especially required for NADPH oxidase dependent ROS generation upon cell activation, that occurs with infectious and inflammatory stimuli, to enable high NADPH yield (132). Upon neutrophil activation here, PPP intermediates accumulate profoundly while many intermediates in glycolysis increase to a lesser extent, indicative of metabolic adaptation by neutrophils (132). NADPH oxidase, the primary source of neutrophil ROS production, oxidizes cytosolic NADPH to reduce molecular oxygen to the superoxide anion (O2^-^) that can metabolize into more potent ROS for effective pathogen clearance (133, 134). The role of PPP for NADPH and subsequent NADPH-oxidase ROS production has been validated. In this study, two chemical inhibitors LDC7559, and NA-11, are both shown to activate the glycolytic enzyme phosphofructokinase-1 liver type (PFKL), the main phosphofructokinase-1 in immune cells, and dampen flux through the PPP in human peripheral blood neutrophils. This inhibits NADPH-oxidase ROS production, NETosis, efficient bacterial killing and tissue damage in an *in vitro* model of acute respiratory distress syndrome (ARDS) (130, 132).

#### 5.2.2 Glycolysis and the electron transport chain

Lectins localized on the bacterial surface bind to carbohydrate-containing epitopes (fucose and mannose glycans) on neutrophil surface to induce intracellular ROS production (135). In addition, neutrophils respond to disseminated candidiasis through enhanced glucose uptake and glycolysis. Glycolysis is elevated through an accumulation of glucose transporter 1 (Glut1) on cell membrane to regulate phagocytosis, ROS production, and fungi elimination (136). Here, neutrophil-specific deletion of Glut1 does not affect neutrophil development, but rather antifungal activity and the control of systemic fungal infection through compromised glucose uptake and glycolysis (136). Moreover, BM derived neutrophils (ckit^+^) appear to be dependent on both glucose metabolism and mitochondrial function for NADPH oxidase dependent ROS production (47, 104). In these studies, inhibition of glycolysis results in a substantial increase in mitochondrial oxygen consumption rate (OCR – measure of ROS production). Inhibition of mitochondria following restricted glucose utilization, completely abolishes OCR (47). On the contrary, inhibition of mitochondrial function in primary murine BM neutrophils with rotenone and antimycin A (complex I and III inhibitors, respectively) alone does not affect early ROS (47). However, reductions in the late phase of the response are seen, suggesting that while the mitochondria is not required for the rapid initiation of neutrophil responses, they may be required to sustain these responses (47). Furthermore, the same study provides evidence that when glycolysis is inhibited by 2- deoxy-D-glucose (2DG) as in cancer, the mitochondria can prioritize ROS production by sustaining levels of NADPH through fatty acid metabolism (**Figure 3**) in the immature, ckit^+^/CXCR2^+^ BM neutrophil phenotype (31, 47, 134, 135). This study also highlights the role of neutrophil phenotypic and functional heterogeneity for the regulation of disease state outcomes.

Other studies show that mitochondria-derived ROS (mitoROS) by the electron transport chain can indirectly regulate neutrophil activation by promoting degranulation and activation of NADPH oxidase upon cell stimulation with fMLP. This emphasizes that on its own mitoROS is inefficient for neutrophil functional responses and that NADPH oxidase is required for efficient microbial killing (137, 138). Inhibition of complex I or III of the respiratory chain upregulates mitochondria ROS production, diminishes LPS-stimulated neutrophils, and reduces severity of LPS-induced acute lung injury in mice (139, 140).

#### 5.2.3 Metabolic perturbations implicated in NADPH-oxidase dependent ROS production

Mitochondrial dysfunction plays an important role in metabolic diseases such as diabetes. Autophagy is shown to be important for neutrophil function as neutrophils from streptozotocin-induced diabetic rats where high incident of infections is prevalent, show autophagy impairment (141). Consequently, reduced ROS production, depolarization of ΔΨmt , low ATP content, and high content of cleaved caspase 3 (apoptosis marker) after phorbol myristate acetate (PMA) stimulation is seen. This is consistent with findings where *Atg7* and *Atg5* knockout mice show impaired degranulation of primary, secondary and tertiary granules and NADPH oxidase ROS (137, 141). Similarly, in another study, phagocytosis and bacterial killing is reduced in *Atg7* and *Atg5* deficient neutrophils (7). This suggests that while mTOR activity, which regulates glycolysis, is enhanced in diabetic rats, optimal neutrophil functions are achieved through mitochondrial metabolism. Here, treatment with metformin and induction of autophagy with AMP-activated protein kinase (AMPK) improves neutrophil responses (111, 125). In acute myocardial infarction (MI), the negative consequences, i.e., tissue damage and heart failure, are attributed in part to upregulation of neutrophil mitochondrial ROS production, and p47 phosphorylation with energy derived from glycolysis (142). However, treatment with arjunolic acid (AA), an antioxidant, reduces ROS via the inhibition of p47 and reduced mitochondrial and glycolytic oxidative burst activity to alleviate the negative consequences of MI (142). *In vivo* models of acute lung injury show that both metformin (used frequently in the treatment of diabetes) and rotenone, can inhibit complex I of the electron transport chain (ETC). This inhibition increases intracellular ROS production by mitochondria, despite obvious reductions in ATP levels, and diminishes production of pro-inflammatory cytokines to mitigate disease severity (134). The mechanism through which neutrophils can use alternative fuel sources in diverse altered nutrient conditions remains to be explored further.

## 6 Metabolic requirements for NET formation

### 6.1 Overview of NETs

Within neutrophils, the ROS released by the NADPH oxidase complex may also induce the formation of neutrophil extracellular traps (NETs). NETs are decondensed chromatin strands of nuclear and mitochondrial DNA (mtDNA), decorated with antimicrobial molecules from primary granules (MPO, NE). These are released into the extracellular space to effectively trap and eliminate large pathogens, such as *Candida albicans* hyphae, *S. aureus*, *K. pneumoniae*, and *P. gingivalis* to name a few, but not small or single microbes, to prevent dissemination of these pathogens to systemic organs (143, 144). NETs generally result in neutrophil death, but NADPH oxidase-independent mechanisms, i.e., mitoROS-dependent, non-lytic form of NET formation also exist, for the degradation of gram-negative and positive bacteria (145). The pathway choice is largely dependent on the specific agonists encountered by the neutrophils (146). Pathogen size sensing by dectin-1 on neutrophils and NE translocation to the phagosome or nucleus, promotes phagocytosis and NET release with small, and large microbes, respectively, for efficient pathogen clearance, minimal tissue damage, and host survival. Lack of dectin-1 or Mpo in mice leads to increased NET formation and local and systemically disseminated *C. albicans* infection (144). However, the origin and mechanism of the DNA scaffold in *in vivo* NETs in bacterial infections remains debated. Transcription factor and kinase (p38 MAPK) activity, peptidylarginine deiminase 4 (PAD4) mediated histone citrullination or ROS-mediated DNA oxidation and repair machinery and resulting chromatin decondensation for NET formation may be important in homeostasis and inflammatory disease states (146–149). In cancer settings, NET formation drives metastases and during sterile conditions, increased NETs are indicative of autoimmune disease states (80, 150). The presence of ‘adherent’ NETs, extruded DNA adhering to the vasculature, by “fresh” neutrophils enhances vascular damage in mice (10). Therefore, despite their role in host defense, excessive NET formation is associated with pathology. As such, blunted NET formation may help to protect mouse and human lungs from exacerbated injury in pneumonia (10).

### 6.2 Metabolic requirements for pathogen killing via NETs

NET formation is strictly dependent on glucose through the glycolytic pathway [**Figure 3**; (136)]. *In vitro* assays show that neutrophils stimulated with 100 nM PMA in glucose-free media exhibit chromatin decondensation within a few hours, resembling the early stages of NET formation, but no NET release. The addition of glucose at a later time point allows for NET formation to occur within 10 minutes (151). Addition of glycolysis inhibitor 2-DG also completely inhibits NET release, while the addition of oligomycin also inhibits the formation of NETs through an undefined mechanism (151). Both NADPH oxidase ROS and autophagy play debated roles in chromatin decondensation for NET formation (152). For example, in trauma patients the increased AMPK activity modulates autophagy and impairs aerobic glycolysis and NETosis causing neutrophil dysfunction and adverse clinical outcomes (121, 153). The role of mTOR and H1F-1a as regulators of NET formation upon LPS stimulation is identified but the mechanism through which H1F-1a regulates NET formation remains to be explored (153). In this case, pharmacological and genetic H1F-1a knockdown reduces NETosis (153). Furthermore, both NADPH-oxidase dependent and independent NET formation by human and murine blood neutrophils are shown to depend on glycolysis dependent lactate formation where inhibition of lactate dehydrogenase (LDH) activity inhibits NETosis (154). The role of glycans, including heparan sulfate, and enhanced glycolysis for NETosis has also been studied extensively (155, 156). The findings presented here suggest that glycolysis, regulated through multiple pathways, plays an undisputed role in regulating maximal neutrophil responses. Here, neutrophil dysfunction following acute trauma is a result of reduced glucose uptake and metabolism. On the contrary, studies show that immature low-density neutrophils (iLDNs; neutrophil precursors) are able to promote breast cancer liver metastasis by executing NETosis under glucose-deprived conditions through glutamate and proline catabolism for mitochondrial-dependent ATP production (80). Furthermore, mitochondrial, optic atrophy type-1 (OPA1), appears indispensable for NOX-independent NET release and bacterial killing through indirect regulation of glycolysis (**Figure 3**) (81, 113, 153). Other agonists known to induce NET formation such as PMA produced similar effects as NETs of mtDNA origin, but the mechanism is unknown.

## 7 Expression of inflammatory mediators by neutrophils

### 7.1 Overview of cytokine and chemokine generation by neutrophils

Expression of cytokines and chemokines by myeloid and non-hematopoietic cells in response to bacterial components via TLR/NF-kB and MyD88 signaling drives neutrophil activation, and inflammation. Neutrophils themselves are capable of generating pro-inflammatory cytokines such as IL1a, IL1β, TNF and IL6 and the chemokines CCL2 and CXCL2, in response to stimulation (2, 9, 128). The TF JUNB is identified as a major regulator of the expression of these mediators (9). The aberrant production of these mediators can also drive pathological destruction of tissues (9, 41, 42).

### 7.2 Metabolic requirement for cytokine and chemokine generation by neutrophils

Studies examining the metabolic requirements for cytokine and chemokine generation by neutrophils in homeostasis and disease states are currently limited. In sepsis, neutrophils are crucial components of the innate immune response and the cytokine storm that characterizes the acute phase response, and this process requires glycolysis (157–159). Inhibition of glycolysis with metformin and rapamycin diminishes survival of mice with sepsis caused by *C. albicans*, lowers *ex vivo* cytokine production (of TNF and IL-1 β ) and increases fungal growth. Therefore, pro-inflammatory cytokine production appears to be a glycolysis driven process whereby stimulated mature neutrophils in inflamed tissues directly generate these inflammatory mediators (**Figure 3**) (9). In cancer, heterogenous neutrophil subsets indirectly influence cytokine production. In fact, a more glycolytic neutrophil (ckit^-^CXCR2^+^ phenotype) is unable to induce T cell death and inhibit interferon-γ (IFN-γ) production by T-cells, thereby enhancing anti-tumor immunity (47). On the contrary, oxidative neutrophils (ckit^+^CXCR2^-^ phenotype) in the tumor microenvironment suppress anti-tumor immunity through IFN-γ (47).

## 8 Inherited metabolic disorders that affect neutrophil function

A total of 485 inborn errors of immunity (IEI) with altered molecular, cellular, and immunological mechanisms contribute to our understanding of the inheritable monogenic defects for immunological disorders. IEI are categorized (160) by the International Union of Immunological Societies Expert Committee. Here, particular gene defects, characterized as combined immunodeficiencies, and congenital defects of phagocyte number or function, relate specially to neutropenia, neutrophil dysfunction and recurrent infections. We do not discuss all the inborn neutropenia and neutrophil disorders in this article. We only focus on those that link inherited metabolic defects with neutrophil dysregulation as metabolic processes are critical for neutrophils’ functional fitness. Neutrophils are capable of glycogen cycling through glycogenesis and gluconeogenesis for energy production and maintenance of effector functions (161). Here, the enzyme glucose-6 phosphatase catalytic subunit 3 (G6PC3) hydrolyzes glucose-6-phosphate to generate glucose for glycolysis and downstream metabolic processes. Neutrophils from hypoglycemic patients with glycogen storage disease type Ib (GSD-Ib) and G6PC3 deficiency in severe congenital neutropenia have impaired ROS, killing defects, and recurrent infection (162–164). This emphasizes the indispensable role of glycolysis and PPP for neutrophil effector functions and infection outcomes. Here, dietary glucose and gene therapy holds therapeutic potential to restore neutrophil dysfunction (165). Similarly, deficiency of any of the oxidase components, due to mutations of autosomal or X-linked recessive genes encoding the 5 components, and the resultant inability to oxidize NADPH results in chronic granulomatous disease (CGD). Neutrophils from these patients migrate and phagocytose normally but fail to generate an NAPDH-oxidase dependent oxidative burst with the resultant failure of intracellular killing leading to recurrent bacterial and fungal infections, including pneumonia (134, 145). The literature on these inherited disease states is reviewed more extensively elsewhere (166) which readers are referred to. Mitochondrial respiration is known to maintain neutrophil differentiation and functions. Pearson syndrome is a rare mitochondrial disorder accompanied with large-scale mtDNA deletions, severe defects in erythroid and myeloid precursor cells, neutropenia, and both severe and fatal infections (167, 168). This emphasizes the previously underappreciated role of mitochondria in neutrophil development and control of infection outcomes. Additionally, 3-Methylglutaconic aciduria due to CLPB, an enzyme for leucine degradation, deficiency is also associated with mitochondrial dysfunction and altered neutrophil differentiation (160). Patients with mutated OPA1 develop autosomal dominant optic atrophy (ADOA) and neutrophils from these patients have a reduced ability for extracellular DNA released and impaired ability for pathogen killing due to impaired complex I activity, and limited NAD^+^ for glycolytic ATP production (113).

## 9 Regulation of neutrophil functions in various nutrient environmental conditions

**Figure 4:**
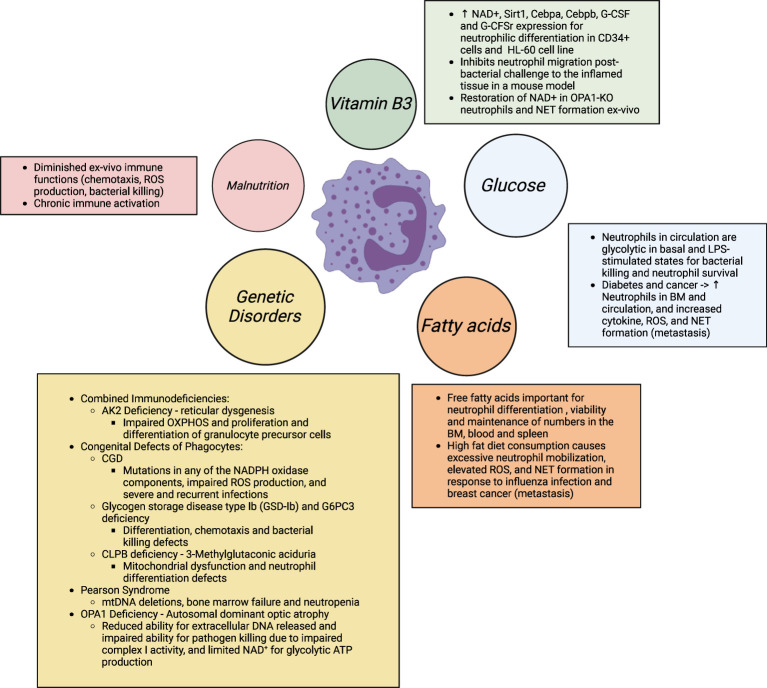
Neutrophil differentiation and functions are disrupted with altered nutrient availability, as well altered usage in multiple genetic diseases. In obesity and associated co-morbidities (diabetes, cancer, chronic inflammation), excess nutrient availability, i.e., glucose and free fatty acids, has shown to prime neutrophils for elevated ROS and pro-inflammatory cytokine production, degranulation, and NETosis for elevated tissue damage. Studies further show that elevated NETs and the metabolically flexible neutrophil subset in obese hosts (immature low- density neutrophils; iLDNs) makes the host more prone to breast cancer cell lung and liver metastasis and adverse outcomes in cancer (8, 169, 170). In contrast, in malnutrition, defined with scarce substrate availability, especially of amino acids, neutrophils are unable to kill pathogens and therefore, have a sustained hyperinflammatory response that leads to morbidity and mortality in this vulnerable population. Similarly, in a myriad of inherited immunodeficiency diseases, altered substrate utilization also predisposes the host to severe recurrent and fatal infections. The IEI classification is based on published literature (156). Supplementation with NAD+ precursors may provide therapeutic benefit in these conditions of neutrophil dysfunction.

Nutrition orchestrates cellular metabolism. Fluctuations in nutrient availability, i.e. nutrient excess and deficiency can modulate metabolic processes and consequently cause hyperactivation or immunosuppression of neutrophils associated with diverse inflammatory disease states (**Figure 4**). Inadequate and inefficacious immune responses are common in more affluent and developed countries with excess nutrient intake (171). Nutrient deficiency is associated with exaggerated inflammation and impaired resolution of inflammation (172, 173).

### 9.1 Micronutrient-dependent regulation of neutrophils in homeostasis and inflammation

#### 9.1.1 Overview of micronutrients involved in neutrophil biology

Different micronutrients relate to neutrophil biology in homeostasis and disease states (174), in particular, Vitamin C (175–178), iron (179–181), zinc (182–185), Vitamin D (186, 187) and Vitamin A (188, 189). In general, a deficiency in any one of these micronutrients is associated with dysregulated neutrophil functions, particularly neutrophil recruitment, degranulation, ROS formation and NETosis, resulting in higher susceptibility to recurrent and severe infections. We particularly focus on the rapidly evolving field of nicotinamide and nicotinic riboside, which are both forms of Vitamin B3 and central mediators of metabolic processes, for the regulation of neutrophil biology.

#### 9.1.2 Overview of NAD and vitamin B3 metabolism

Nicotinamide adenine dinucleotide (NAD^+^) is an essential cofactor that plays an indispensable role in key redox reactions and serves as a substrate for PARPs, sirtuins, CD38, ADP-ribosyl-transferases, sterile alpha and TIR-motif-containing protein 1, and RNA polymerases. For its role in oxidation-reduction reactions, it accepts electrons from glycolysis, FAO and TCA intermediates to form NADH and feeds them into complex I of the electron transport chain for OXPHOS. As such, NAD^+^/NADH are essential regulators of many cellular processes. Despite its fast and dynamic metabolism, NAD^+^ concentration reflects the balance between NAD^+^ consumption and synthesis from the *de novo*, Preiss-Handler and salvage pathways. The *de novo* biosynthesis of NAD results from the breakdown of tryptophan mostly in the liver (190). However, the major pathway of NAD^+^ biosynthesis is the salvage pathway. Here, NAD^+^ is converted to nicotinamide (NAM), a form of vitamin B3, and reconverted to NAD^+^ using the enzyme nicotinamide phosphoribosyltransferase (NAMPT). Nicotinamide riboside (NR), another form of vitamin B3, can also enter the salvage pathway through NR kinase (Nrk1/2)-mediated phosphorylation of NR into nicotinamide mononucleotide (NMN) (99). Progressive decline in NAD^+^ homeostasis and mitochondrial function are common hallmarks of ageing and disease pathologies including neutrophil dysfunction and hyperinflammation. While some studies report beneficial effects of NAD^+^ precursors such as NR and NAM to counter age-related functional decline in murine models, others report no such effects in human subjects [reviewed in (191)]. We focus on the role of NAD metabolism in neutrophil biology.

#### 9.1.3 NAD and vitamin B3 metabolism in neutrophils

The treatment of hematopoietic progenitor (CD34^+^) cells in healthy individuals with either G-CSF or NAM increases intracellular levels of both NAMPT and NAD^+^. Neutrophilic differentiation is also induced through NAD+-dependent sirtuin-1 activation, subsequent binding and activation of Cebpa and Cebpb, and, ultimately, upregulation of G-CSF synthesis and G-CSF receptor expression (91). NAMPT expression is highest in promyelocytes and NAMPT is specifically localized to the mitochondria (91, 192). This suggests that neutrophil precursors express functional mitochondria for NAD^+^ conversion from NAM and therefore neutrophil differentiation regulation via the mechanism that is proposed (91). However, no study has studied this mechanism in closer detail.

NR supplementation is shown to improve HSC and progenitor function by increasing mitochondrial clearance in HSCs. This finding is surprising since multiple publications consistently show an increase in mitochondrial fitness with NR treatment (99, 193, 194). Nevertheless, no study has yet examined how NAD^+^ precursor treatment in committed progenitors affects differentiation where mitochondrial function is preferred to sustain increased ATP demands. While an increase in granulocytic differentiation is shown in steady-state through the involvement of NAMPT, NAD^+^, G-CSF and G-CSFR upon NAM treatment, the role of NAM on NAD^+^ in regulating neutrophil homeostasis in steady-state and emergency granulopoiesis needs clarification. Oral NR supplementation at a dose of 400 mg/kg/day for 8 weeks shows an alleviation of the BM HSC ageing phenotype, that is normally dominated by a significant expansion of the HSC pool and differentiation skewed towards the myeloid lineage and increased metabolic activity of HSCs. NR promotes molecular and mitochondrial changes in the aged BM that resembles the young HSC state including improved NAD/NADH ratio, reduction in the frequency of myeloid and lymphoid progenitors, improvement in age-deregulated HSC genes, and a reduction in the metabolic potential of aged HSCs at the transcriptional level accompanied with increased sirtuin-3, for mitophagy. Here, there is a modest restoration of the ageing HSC phenotype with NR but no effect of NR is observed in the differentiated myeloid cells (26). Moreover, increased extracellular NAD^+^ in inflammatory conditions can help delay mitochondrial-dependent neutrophil apoptosis at inflamed sites (169). This is dependent on NAD^+^ inhibiting the degradation of Mcl-1 (anti-apoptotic), suppressing Bax translocation to the mitochondria, attenuating the dissipation of mitochondrial membrane potential and cytochrome C release from mitochondria into the cytosol and supressing caspase-9 and -3 activation (169). The role of administrating 1000 mg/kg NAM in attenuating exacerbated neutrophil recruitment to inflamed tissues in murine models has also been studied although the downstream molecules regulating this process has not been determined (195, 196). NAM and nicotinic acid effectively inhibit neutrophil migration when administered twice, i.e., 30 minutes before and 1 hour after bacterial insult while single administration after bacterial challenge fails to prevent neutrophil recruitment into the mouse pleural cavity (195, 196). In mice with OPA1 gene mutations where NAD^+^ is reduced, treatment with NMN rescues systemic NAD^+^ levels. This in turn helps to indirectly regulate NET formation following neutrophil activation (**Figure 3**) (113).

### 9.2 Glucose requirements in neutrophil biology

Neutrophils function in metabolically challenged environments. Human peripheral blood neutrophils are primarily glycolytic in both basal and LPS-stimulated states (161). With glucose deprivation, these cells can maintain their intracellular energy homeostasis provided their glycolytic pathway is active due to increased glycogen stores in these cells (161). Interestingly, neutrophils express enzymes required for active glycogenesis and gluconeogenesis that enables this metabolic flexibility in glucose deprivation and other metabolically challenged environments. Through ^13^C labeling, glutamine is identified as the substrate in gluconeogenesis, where inhibition of these processes and associated enzymes impairs bacterial killing and neutrophil survival, and promotes systemic spread of bacteria in mice (161).

#### 9.2.1 Role of glucose in obesity-associated comorbidities

Hyperglycemia, as is commonly in diabetic mice and humans, is directly associated with an increase in BM and circulating neutrophils with an increased expression of the calprotectin subunit S100A8, and extracellular ROS production (197). Similarly, incubation of neutrophils from healthy donors with high glucose media or neutrophils from diabetic mice produces more PAD4 and NETs for an impaired wound healing response (198). Additional dysregulated functions of neutrophils in type 1 and 2 diabetes have been reviewed elsewhere (199). The mechanisms by which increased glucose availability alters neutrophil biology, for the heightened inflammatory response and tissue damage has not yet been addressed. Persistent elevation of glucose levels in diabetes and polyol and hexosamine pathway may be involved but this has not been studied mechanistically in the forementioned studies and should be the focus of future studies.

### 9.3 Fatty acid metabolism in neutrophils

Endogenous fatty acid synthesis and utilization is crucial for the maintenance of mature neutrophils. Dietary lipid intake in neutrophil-specific fatty acid synthase (FAS) knockout mice can partially reverse the aberrant phenotypes of FAS deficiency, reduced neutrophil viability and numbers in the BM, blood and spleen (200). Similarly, in *Atg7* deficient murine myeloblasts, pyruvate treatment or exogenous free fatty acids (linolenic acid or a mix of unsaturated and saturated free fatty acids) alone is sufficient to restore normal glucose metabolism and rescue the defective neutrophil differentiation.

#### 9.3.1 Role of free fatty acids in obesity and associated comorbidities

High fat consumption is linked to poor outcomes of influenza infection in BALB/c mice fed a high-fat diet (HFD) for 18-weeks due to elevated H_2_O_2_ concentration and NET formation (201). Additionally, mice on the HFD for 15 weeks injected with breast cancer (BC) cells have lung neutrophilia, and increased lung vascular permeability compared to mice fed an isocaloric low-fat diet. This is attributed to higher NADPH-oxidase dependent ROS, granule proteins, pro-inflammatory cytokines and NETosis by lung neutrophils from obese hosts. Therefore, BC cells in the lungs (lung metastasis) of obese mice are increased compared to lean mice. In this regard, depletion of neutrophils with anti-Ly6G antibody in obese mice reduces lung permeability and BC cell extravasation (8, 202). Similarly, obese human subjects with elevated BMI (35–68 kg/m2) and serum triglycerides have elevated chemotaxis and superoxide generation in unstimulated and stimulated neutrophils compared with lean controls. In this case, phagocytosis is not affected (203).

### 9.4 Protein and amino acid metabolism for neutrophils’ biological processes

The role of key amino acids such as glutamine on neutrophil biology has been discussed above. In this section, we discuss the impact of perturbed amino acid and protein homeostasis for neutrophil biology. In chronological aging, alterations in protein intake and anabolic metabolism, especially of the essential branched-chain amino acid, leucine, is associated with age-related progressive loss of muscle mass, osteoporosis and frailty, and can be overcome with a higher level of protein intake (204–206). In this context, increased neutrophil numbers and degranulation, reduced coordinated neutrophil migration, phagocytosis, ROS production and NET generation, and a heightened pro-inflammatory state contributes to loss of skeletal muscle mass [reviewed in (207–209)]. Particularly, an expansion of neutrophils with a capacity for reverse transmigration and increased remote organ damage has been identified (41). Additionally, various basic leucine zipper TFs constitute the transcriptional network for the neutrophil life cycle of which, leucine content is a major determinant. However, no study to date has yet explored how dysregulation of specific amino acids, such as leucine, influences neutrophil biology in aged tissues. A randomized clinical trial has assessed the effects of whey protein and leucine ingestion post-exercise on neutrophil functions, which are attenuated during intensive and prolonged endurance exercise, during 6 days of intense cycling in 12 male cyclists (210). Leucine-enriched whey protein ingestion improves neutrophil oxidative burst post-exercise, to prime neutrophils for host immunity and tissue repair (210). Additionally, the effects of a hypocaloric Mediterranean diet (MD) and two high protein diets, with (HPW) and without (HP) whey protein supplementation, on body composition, lipid profiles, inflammation and muscle-damage blood indices in overweight, sedentary, young participants has been assessed (211). Neutrophil-mediated inflammation and muscle-damage is increased in HP and HPW compared to the MD group. No other aspects of neutrophil activity or functions are studied here (211). The association between dysregulated tryptophan-kynurenine metabolism and neutrophils in obesity is also summarized extensively (212).

#### 9.4.1 Nutrient deficiencies as contributors to neutrophil dysfunction

Earlier, we focus on excess intake of macronutrients as they relate to co-morbidities of obesity including diabetes, cancer, and chronic inflammation. Multiple excellent articles and reviews have already been published on this in great detail that readers are referred to for additional learning [reviewed in (213–216)]. The section below is focused on inadequate nutrient intake as it relates to undernutrition and neutrophil perturbations, on which very little has been published to date.

In undernutrition, referred to as malnutrition in this section, inadequate intake of protein and calories, and micronutrient deficiencies predisposes children to diminished immune functions and chronic immune activation that increases susceptibility to infections and multiple organ failure [reviewed in (173, 217–219)]. There are limited studies performing functional assays of immune cells in low-and-middle-income countries in children with varying forms of malnutrition. Some longitudinal studies compare immune cell function in children with severe malnutrition at admission, and during recovery with exclusion of HIV-positive children. Even more limited studies in childhood malnutrition suggest some impairments in peripheral blood neutrophil function. Here, *in vitro* assays show reduced chemotaxis in children with severe malnutrition but no infection (220, 221). In another context, Leishmania donovani, a parasite that causes visceral leishmaniasis, in children with malnutrition reduces *in vitro* neutrophil and monocyte TEM after PMA stimulation as determined indirectly by flow cytometry using CD62L (222). The same study also shows reduced ROS production in these cells as determined by Dihydrorhodamine 123, but the study did not stratify by the degree of malnutrition in the sample (222). In children with severe malnutrition but no infection, *in vitro* microbicidal defects are shown to occur with *S. aureus*, *E. coli* and *C. albicans* in mononuclear and polymorphonuclear cells, while the postphagocytic morphological events, including vacuole formation and degranulation, are normal (220, 221). The data on phagocytosis is inconsistent, with some reporting unaffected phagocytosis whist others observing reduced phagocytosis especially when infection is present (217, 219, 223–225). Similarly, consensus points to impaired ROS production determined through nitroblue tetrazolium reduction assay and impaired bacterial killing capacity in children with severe malnutrition with or without infection (217, 224–227). It is unknown from these assays however whether NADPH or mitochondrial ROS production is hindered in malnutrition. These studies are conducted in different geographical sites, and differing disease states which may underlie the inconsistencies in findings. While the data suggests that not all functions are affected to the same extent, these studies only show associations and not the causal pathways linking malnutrition to impaired functions and clinical outcomes. Mice fed a protein-free diet have less neutrophils in infected lungs and this reduction could be related to impaired granulopoiesis, but the mechanism is not elucidated (224, 228). On the contrary, increased immature neutrophils are found in the blood defined through electron microscopy and flow cytometry in human cohorts while others report no change in total blood leukocyte count (220, 226, 229).

### 9.5 Therapeutic strategies for neutrophil recovery in nutritional deficiencies and excess

Anorexia, characterized by phenotypes and increased susceptibility to bacterial pathogens observed in undernutrition, is also an evolutionary conserved common response to infectious diseases. Immunological changes reported in anorexia are reviewed elsewhere in greater detail (230). Here, the refeeding syndrome is a serious complication of anorexia treatment that results from excessive nutrient supplementation resulting in rapid hormonal and metabolic disturbances and high morbidity and mortality (170, 231). In fact, diminished ROS production during the initial period of refeeding (11-40 days of hospitalization) in patients with anorexia that return to normal values to that of healthy control subjects with an extended period of refeeding is commonly seen (232). A fiber-rich diet in this refeeding regime may be sufficient to restore efficient immunity (233).

In conditions of nutrient excess, inadequate and inefficacious immune responses also underlie poor infection outcomes, as discussed above. This includes exacerbated neutrophil mobilization, and ROS and NET formation that prolongs inflammation in obesity (8, 216). Here, mice fed a mild calorie restricted diet regimen had an improved pulmonary anti-mycobacterial host response with reduced bacterial load, and lung immunopathology (172). In addition, adiponectin, a hormone that is depleted in obesity, repletion in obese mice and humans regulates neutrophil oxidative burst through its anti-inflammatory properties (234). Clinically approved drugs for type 2 diabetes including pioglitazone (235), a PPARɣ agonist and metformin, can induce adiponectin in mice and humans to regulate the exacerbated inflammatory responses in this context. How these therapeutics directly target immune cell metabolism remains to be studied further, but modulation of autophagy and mitochondrial metabolism may be important here (236). Gout is a debilitating chronic inflammatory arthritis, exaggerated by age and diet-induced lipotoxicity. Here, a ketogenic diet ameliorates neutrophil IL-1β secretion by increasing levels of β-hydroxybutyrate and inactivating NLRP3 inflammasome to protect the mice against inflammation (237, 238). Through its histone deacetylase inhibitory activity and therefore gene expression, β-hydroxybutyrate also regulates neutrophil-mediated immunity (233, 239). As a result, this therapeutic strategy is also relevant for other inflammatory diseases related to nutrient excess that are driven by chronic neutrophil activation.

## 10 Concluding remarks & perspectives

In the last decade, progress has been made to discern the metabolic requirements and flexibility of neutrophils that extends beyond glycolysis using traditional and novel immunometabolism approaches. Importantly, we discuss in this review from evidence in humans and mouse models reviewed here that neutrophils rely on the activity of multiple metabolic pathways to fulfill their energy requirements throughout their life stages. Hindrance in these processes, substrate utilization and breakdown, disrupts optimal neutrophil biology. Here in steady-state, neutrophil committed progenitors rely on fatty acid oxidation and mitochondrial ATP generation for differentiation while mature neutrophils require both mitochondrial and non-mitochondrial sources for energy production. In inflammatory states including obesity, and undernutrition, the ability to sense and reprogram metabolism based on nutrient availability also marks the response in these conditions. In these contexts, heterogenous neutrophils populations with different energy requirements may be recruited to mediate the inflammatory response and clinical outcomes, although this remains to be explored further. By further understanding the unique role of metabolism, although not ‘black and white’ for neutrophil biology, we will be better able to therapeutically modulate these pathways for better outcomes in inflammatory disease states.

## 11 Outstanding questions

Despite advancement in the understanding of neutrophil metabolism with newer technologies, much more work needs to be done to uncover the molecular mechanisms coordinating the neutrophil lifecycle and antimicrobial functions before we fully understand neutrophil metabolism for its modulation in different disease contexts. Here, we provide few examples of key areas where additional work is needed.

### 11.1 HSC and neutrophil development in the BM

Although HSCs are early determinants of neutrophil commitment, we still do not know how the cellular and metabolic components of HSCs interact, either directly or indirectly, to regulate neutrophil development and functions under steady-state and stress conditions. The mechanism linking NAD^+^ supplementation with regulation of TFs for neutrophil differentiation and maturation (Cebpa, Cebpb) has not been addressed to date and remains to be studied. Autophagy, mitochondrial respiration, and their link with gene regulation remains to be studied in the human neutrophil developmental trajectory.

### 11.2 Neutrophil migration

Newer technologies provide a better understanding of the metabolic requirements for efficient neutrophil migration in healthy tissues. Therefore, investigating neutrophil migration behaviour in inflammatory pathology and metabolic challenge may have implications in the resolution of human disease where neutropenia or neutrophilia is common. The metabolic requirements for TEM and swarming also remain unexplored and require further investigation.

### 11.3 NADPH oxidase ROS production

There is evidence that glycolysis regulates NADPH oxidase activity, but we still do not understand how glycolytic activity regulates NADPH oxidase subunits’ activation and the mechanism that connects reduced glucose utilization to enhanced mitochondrial capacity in inflammatory disease states. Here, the role of metabolites in controlling chromatin modifications, DNA methylation, and post-translational modifications of proteins for the determination of cell fate and function will be relevant, but this has never been explored for neutrophil biology. The advent in the integration of multiple single cell ‘omics’ enables for such research and might be worth exploring in this context. Additionally, the mechanism of the interplay between neutrophil metabolism, differentiation and ROS-mediated functions requires further investigation in homeostasis and disease states to aid in the development of targeted therapeutics.

### 11.4 NET formation

It was highlighted in the text that NET formation requires autophagy and while autophagy may play a role in removing damaged mitochondria, mitophagy, the exact mechanism through which autophagy regulates NET formation remains unclear and requires further clarification. Accumulation of lactate and NETs may partially explain disease severity and adverse outcomes associated with sepsis, however how lactate can trigger NETosis remains elusive (154). Although H1F-1a regulates NETosis, optimal NETosis also requires NADPH oxidase ROS, which is decreased under hypoxia due to limited oxygen availability. It would therefore be interesting to explore the extent to which H1F-1a can help recover NETosis in hypoxia where NADPH oxidase ROS is limited. Additionally, the role of mitoROS for NOX-independent vital NET release in this mechanism remains disputed and requires more exploration.

### 11.5 Neutrophil crosstalk with other cells

Studies are needed to examine metabolic programming involved directly in neutrophil communication with immune and non-immune cells irrespective of the soluble mediators that have often been discussed previously.

### 11.6 Neutrophils in altered nutritional environments

As decline in NAD^+^ has been implicated in many diseases, future studies must investigate the impact of changes in NAD^+^ availability on innate immune responses, specifically that of neutrophils and the mechanisms involved in the dysregulation. Additionally, studies must investigate the mechanistic pathway regulating granulopoiesis and neutrophil functions with the supplementation of NAD^+^ and its precursors’. Studies have also not yet addressed how free fatty acids regulate epigenetic modifications to drive neutrophil differentiation, irrespective of mitochondrial ATP generation. The molecular mechanism through which excess glucose and metabolic rewiring in diabetes promotes neutrophilia and exacerbates inflammatory disease requires further exploration in mice and humans. This is crucial in the context of diverse tissue microenvironments where excess circulating glucose is accompanied by overt pathologies in multiple organ systems. Similarly, it is not known if correction of circulating glucose levels in these conditions eliminates neutrophil dysfunction and associated pathologies. Additionally, how excess glucose alters neutrophil phenotype and function needs to be studied further. Future research needs to focus on the exploration of novel neutrophil-centered treatments to resolve inflammation and improve patient outcomes in conditions of excess glucose availability. The causative mechanism of fatty acid excess on neutrophil functional diversity and inflammation has not been identified. Studies mentioned in the *Role of Free Fatty Acids in Obesity and associated Comorbidities* section suggest that although free fatty acids are crucial for normal neutrophil functions, excess substrate availability promotes the emergence of functionally distinct neutrophils that can promote aberrant disease states. More mechanistic studies are needed to evaluate the effects of varying quantities of fatty acid consumption on neutrophil biology. The existing studies with the existent pre-clinical models do not accurately represent phenotypic changes associated with severe malnutrition where multiple physiological changes are observed therefore the translational ability of these studies is questioned. This warrants the need to develop pre-clinical models that are representative of real-life in which to modulate metabolism and improve neutrophilic processes. Additionally, most of the studies conducted in children with malnutrition have major limitations: small sample size, lack of appropriate control groups, i.e., adequately nourished children from high-income counties as controls with no overt infection, and several forms of malnutrition. These studies are also limited by ethical constraints and methodological limitations, i.e., tissue sampling and immunological techniques. The mechanisms exacerbating impaired neutrophil functions in response to malnutrition and infection remains unexplained since observational studies can only determine associations, and not causality (145, 217). In this case, genetic approaches to decipher proposed pathways have not been undertaken and therefore causation cannot be determined. It is also impossible to delineate from these studies whether neutrophil dysfunction is a cause or consequence of malnutrition when infection is present. Additionally, the impact of specific metabolites and metabolic perturbations has not been closely explored in neutrophils from children with malnutrition especially since multiple metabolic derangements exist in this context and the role of limited substrate availability in this case also requires further exploration. Impaired autophagic flux and mitochondrial dysfunction has been associated with hepatic steatosis and gut barrier dysfunction in a mouse model of severe malnutrition (240–242). Differences in blood metabolomic profiles of children with severe malnutrition that died, especially an increase in metabolites of mitochondria-related bioenergetic pathways has also been identified (243, 244). Therefore, the role of mitochondria for neutrophil-associated catastrophe in the malnourished host requires further exploration. Additionally, the impact of specific micronutrient deficiencies on neutrophil biology has not been studied in detail in malnutrition. The impact of refeeding in malnutrition on additional neutrophil functions beyond ROS production and how it contributes to the refeeding complications is not well known. The role of changes in specific macro and micronutrient composition with refeeding and their impact on neutrophil biology is unknown and requires further exploration. It is well-accepted that mitochondrial function is lost as neutrophils mature and heterogeneity in neutrophil metabolic programming exists between different tissues and within neutrophil subsets. As such, uncovering neutrophil metabolic adaptations in inflammatory disease states and across heterogenous neutrophil subsets may help in targeting these pathways to improve long-term outcomes. Similarly, the role that the metabolic switch plays at inflammatory/infectious sites during metabolic disorders for outcome determination requires further investigation. We believe these studies will help novice and expert researchers in the field expand our understanding of the unique role of metabolism in steering neutrophil biology, a field that has largely remained neglected.

## Author contributions

MT: Conceptualization, Formal analysis, Investigation, Visualization, Writing – original draft, Writing – review & editing, Project administration. HU: Writing – review & editing. MG: Writing – review & editing. NP: Writing – review & editing. CB: Writing – review & editing. AG: Writing – review & editing. CL: Writing – review & editing. JB: Writing – review & editing. RB: Writing – review & editing, Conceptualization, Funding acquisition, Investigation, Project administration, Supervision, Resources. AF: Conceptualization, Investigation, Project administration, Supervision, Writing – review & editing, Validation, Writing – original draft.
